# Pittsburgh 1991 Conference – New products

**DOI:** 10.1155/S1463924691000299

**Published:** 1991


Journal of Automatic Chemistry, Vol. 13, No. 4 (July–August 1991), pp. 153–164

### Pittsburgh 1991 Conference – New products

### ELEMENTAL ANALYSERS

### On-line process chemical analysers

A process analysis system (EPAS), from Eppendorf, for the chemical processing industry combines high-speed analysis with performance and reliability. Simplicity of analyser design guarantees long-term reliability and ease of service. Diagnostic software monitors and verifies analytical process data and analyser performance. An extensive process applications library has been developed.

*Eppendorf North America, Inc., 545 Science Dr., Madison, Wisconsin 53711, USA.*

### Automatic fluxer

The FX-503 automatic fluxer provides programmable methods for melting temperature, agitation-direction, amount and speed of agitation over a number of phases. The system can be programmed for methods containing up to 100 phases. Precise temperature and agitation characteristics are controlled by the microprocessor. Flexibility of programming permits use in both peroxide or borate applications.

*LECO Corp., 3000 Lakeview Ave., St. Joseph, Michigan 49085-2396, USA.*

### PARTICLE SIZE ANALYSERS

### In-stream particle analyser

The EPCS particle analyser fits directly into the process stream via flange connection (pipes 6 in, or larger.) The rugged, real-time instrument provides 100% representation data for total quality control. Remote operation reduces employee contact with process machinery and product. Size (general range: 1 to 500 μm) and concentration of irregular or spherical shaped powders can be measured. The flange operates in concentrations up to 1000 g/$m^3$. Slipstream hardware can be designed for higher densities.

*Insitec, Inc., dba Insitec Measurement Systems, 2110 Omega Drive, Suite D, San Ramon, California 94583, USA.*

### Modular particle size analyser

The Microtrac series 9200 particle size analyser measures 0·005 to 700 μm with a modular design, so users can add units as needed. The ultra-fine particle analyser is a submicron instrument that can measure at high concentrations. The full-range analyser covers 0·1 to 700 μm in a single measurement with no parts movement or replacement. The computer control module controls analyses, displays results, and manages past results.

*Leeds & Northrup, Sumneytown Pike, North Wales, Pennsylvania 19454, USA.*

### THERMAL ANALYSERS

### Automated DSC system

TA Instruments launched a fully automated DSC system which allows up to 62 samples to be thermally evaluated and analysed unattended. The operator simply loads an autosampler tray, initiates the system, and returns later to retrieve a stack of hard copy results. The system is suitable for increasing productivity in both R & D and QC laboratories.

*TA Instruments, Inc., 109 Lukens Dr., Wilmington, Delaware 19720, USA.*

### AUTOSAMPLERS

### Organic sampler

The model AVOCS-500 is a self-contained automatic volatile composite organic sampler. The unit is used to obtain and store samples of liquid containing volatile organic compounds for Analytical Chemical Methods No. 624, 1624, 601, 602, 524, and for Test Methods for Evaluating Solid Waste No. SW-846 Methods No. 8240 and 8260. The motor-driven, gas-tight container allows laboratories to collect 100 ml of samples, at 0.21 ml per sample, over a selected period of time. This technique improves the current grab sampling system.

*Associated Design and Manufacturing Co., 814 N. Henry St., Alexandria, Virginia 22314, USA.*

### DSC and TGA autoanalysis software

DSC and TGA autoanalysis software programs offer increased productivity and ease-of-use by allowing the system controller (computer) to analyse data from repetitive experiments, unattended. The system chooses from more than 100 stored routines to automatically analyse either data at the completion of an experiment or data files from up to 10 earlier experiments. The software is sufficiently flexible for both R & D and QC laboratories.

*TA Instruments, Inc. (as above).*

### Syringes and autosamplers

PTA-30 and PTA-30W/S purge and trap autosamplers are for automated soil and water analyses using existing P & T Concentrators. The purge and trap systems are: DynaTrap for waters, soils, air, and VOST; DynaSoils for soils only, and DynaWaters for waters only.

*Dynatech Precision Sampling Corp., P.O. Box 15886, 8275 W. El Cajon Dr., Baton Rouge, Louisiana 70895, USA.*

### Dissolution autosampling system

Hanson Research announced a new model upgrade of its Dissoette Autosampling System for Dissolution Test Applications – the Dissoette II. The upgrade includes an advanced SIP microprocessor controller which accepts program protocol from an IBM PC computer down-load and saves program protocols in non-volatile memory storage. The Dissoette II has been expanded to include automatic UV detection from samples collected in a built-in 144-position carousel, and now offers direct-inject of collected samples into the Waters Millipore 712D Automatic Dissolution/HPLC system for complete dissolution-HPLC automation. Dissoette II features include:

(1) 6 or 12-flask systems.
(2) New memory storage of test protocols.
(3) Factory-programmed; accepts IBM PC down-load programs.
(4) Handles sampling, collection and detector interface.
(5) Automatic media replace in dissolution flasks.
(6) One system can handle both UV and HPLC.
(7) New UV Auto-detect from 144-position collection carousel.
(8) New Auto-detect program includes standard and wash.
(9) Interfaces with most modern spectrophotometers.
(10) UV DATA-PAK software for computer-generated test reports.
(11) Automatic collection into virtually any standard HPLC vial.
(12) New direct-inject into Waters 712D Dissolution/HPLC System.
(13) All Teflon flow system.

*Hanson Research Corporation, 9810 Variel Avenue, Chatsworth, California 91311, USA.*

### HPLC autosampler

The autoMetric autosampler provides reproducibility for virtually any HPLC application requiring unattended sample introduction. The autosampler is a no-loss, variable volume, programmable auto-injection system designed for complete electrical operation, eliminating the need for an accessory gas. A total of 96 different samples can be analysed with an infinite number of injections possible from each sample. Injection sizes from 1μl to 1 ml are possible with all operating parameters programmed through a keypad on the front panel.

*LDC Analytical, 3661 Interstate Industrial Park Rd. N., P.O. Box 10235, Riviera Beach, Florida 33419, USA.*

### Cryogenic preconcentrator

The model 3550 automated TO-14 cryogenic preconcentrator is a microprocessor-controlled instrument. Features include: two distinct flow paths for ambient and source sample introduction, all electronic valves, a TO-14 cryotrap, 16-sample automation, automatic internal standard introduction, and variable loop injection.

*Nutech Corp., 2806 Cheek Rd., Durham, North Carolina 27704, USA.*

### GAS CHROMATOGRAPHY

### Continuous water analyser

The Siemens H-202 continuous water analyser unites a sample

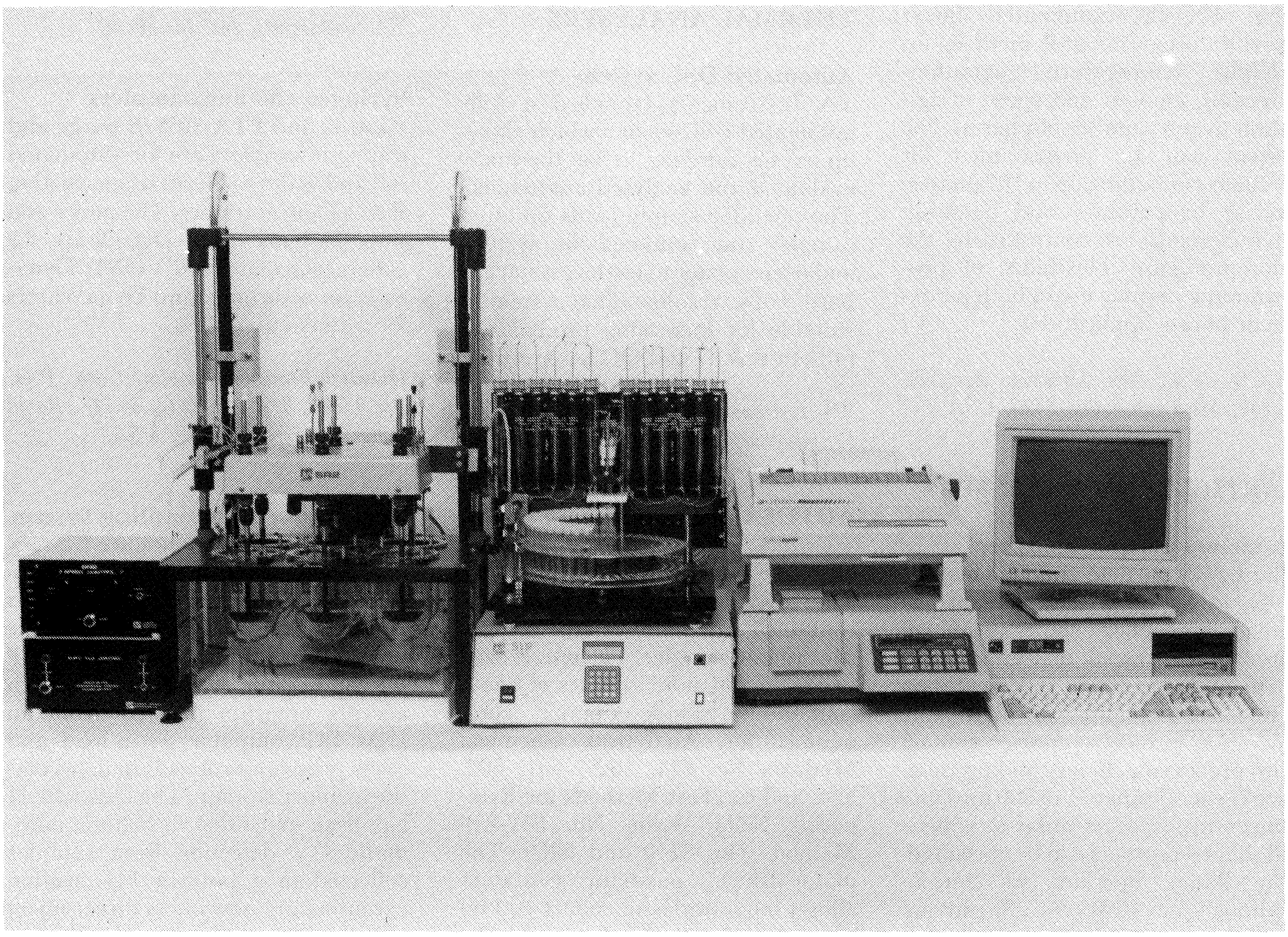

*The Dissoette II Dissolution Autosampling System.*

enrichment sparger with the PGC-102 multidimensional process GC and permits unattended monitoring of water streams for volatile hydrocarbons down to the 1 ppb level. The instrument can monitor influent and effluent of a waste treatment facility, monitor non-contact cooling water, and analyse clean water used in production.

*ES Industries, 8 S. Maple Ave., Marlton, New Jersey 08053, USA.*

### Headspace autosampler

The model 7000 headspace autosampler offers ease of sample preparation and increased overall speed of analysis. With the use of Method Optimization Mode MOM, the user can determine the optimum headspace sampling times, temperatures, and mixing values. Using the OPTI-MIX equilibration system, both thermal energy and pulse mixing energy are simultaneously applied during the sample equilibration process.

*Tekmar Co., P.O. Box 371856, Cincinnati, Ohio 45222-1856, USA.*

### Stream sampling system

Samplivalve is available in corrosion resistant materials of PTFE and Kel-F. With this system, the user can time-share a chemical analyser among 12 different chemical stream samples. The unit can be automatically rotated to select any one of 12 different chemical streams and will divert a sample into the analyser. The 11 streams that are not being sampled are allowed to flow through the unit.

*Scanivalve Corp., 10222 San Diego Mission Rd., San Diego, California 92108, USA.*

### Sampler

The model 501 random access sampler is designed for the Alpkem RFA microcontinuous flow analyser and other automated analytical instrumentation. Under computer control and interfaced with the 511 dilutor, it can resample and dilute off-scale samples automatically. The sampler can also operate as a stand-alone unit. Equipped with an X, Y, Z arm, the probe can access any position on the sample racks and automatically resample check standards at user designated intervals.

*Alpkem Corp., P.O. Box 260, Clackamas, Oregon 97015, USA.*

### Gas sparging gas chromatograph

The Optichrom Advance gas sparging process chromatograph provides complete capabilities in water or wastewater analysis for many of the volatile contaminants described in EPA lists 601 and 602 by coupling a fully automatic sample enrichment system with a process GC. Typical applications include the measurement of volatile organics in process wastewater in the ppb range. The sparging unit consists of a borosilicate glass sparger, pressurization vessels, sample heating compartment, and flow controller.

*Applied Automation Hartmann & Braun, P.O. Box 9999, Bartlesville, Oklahoma 74005, USA.*

### Continuous water monitoring system

The Aquascan system is a static turnkey water monitoring GC system for detecting volatile organic compounds to sub-ppb levels. Totally computerized, the system automatically acquires a water sample and performs a specified number of analysis cycles with preselected periodic autocalibration. Accurate and reliable, the system can operate remotely, storing and transmitting data for proper documentation.

*SENTEX Sensing Technology, Inc., 553 Broad Ave., Ridgefield, New Jersey 07657, USA.*

### Automated concentrator

The model 2000 automated concentrator is an autosampler for the analysis of source and ambient level volatile organic compounds in air or similar fixed gases. The system permits the unattended analysis of 16 samples, and includes such features as self leak checking, manifold back-flushing, on-line pressure monitoring, and QA/QC report generation. Modular traps allow easy replacement and/or rapid conversion from cryogenic to sorbent concentration. Multiple sample concentration configurations are available.

*Entech Laboratory Automation, 950 Enchanted Way, #101, Simi Valley, California 93065-0908, USA.*

### Pyrolysis GC injector

The Pyrojector II is a microprocessor-controlled continuous-mode, constant temperature microfurnace pyrolyzing unit designed to attach directly onto the GC injector. The instrument is supplied with three types of sample introduction systems: septum injecion head for liquid samples, septumless injection head for solid samples, and pelletizer injection head for larger amounts solid samples.

*Scientific Glass Engineering, Inc., 2007 Kramer Lane, Austin, Texas 78758, USA.*

### Dual HPLC/HRGC system

The Carlo Erba Dualchrom 3000 employs both HPLC and HRGC in a single system, allowing direct on-line coupling. A complete turnkey, fully automatic system, the unit is ideally suited for full type and class separation on a wide variety of samples relating to environmental, petrochemical, petroleum, and other applications. The Dualchrom 3000 incorporates the sample preparation stage in the analytical run, thus reducing sample preparation time and manpower.

*Fisons Instruments, 24911 Stanford Ave., Valencia, California 91355, USA.*

### Automated system for pesticide residue screening

A dedicated system for the screening of all types of foods for pesticide residues saves time and money through reduced operator interpretation, increased throughput, and ease of use. The instrument eliminates the need for confirmatory analysis in terms of accuracy and precision in both quantitative and qualitative analysis. The instrument uses simultaneous dual capillary column GC analysis. A software package calculates retention indices and compares the results to those in a user-defined library to identify the pesticide residues to 99·9% accuracy.

*HNU Systems Inc., 160 Charlemont St., Newton, Massachusetts 02161-9987, USA.*

### GEL PERMEATION CHROMATOGRAPHY

### GPC sample processor
A low-cost system for automated sample cleanup by gel permeation chromatography, the microprocessor-based SP-1000 GPC sample processor, allows environmental laboratories to comply with US EPA method requirements for sample cleanup. This technician-level instrument offers simple programming with audible or LED alert for end of processing, user interrupt, or power failure.

*ABC Laboratories, P.O. Box 1097, Columbia, Maryland 65205, USA.*

### LIQUID CHROMATOGRAPHY

### Universal, low-cost, HPLC Autosampler
A universal, low-cost, HPLC Autosampler is now available from Alcott Chromatography, Inc. The Model 738 Autosampler can inject 1 μl to 1 ml from up to 96 different samples without any loss of sample and fits any HPLC or ion chromatograph. Furthermore, the unit can make an unlimited number of injections from any single sample and requires no accessory gases for operation.

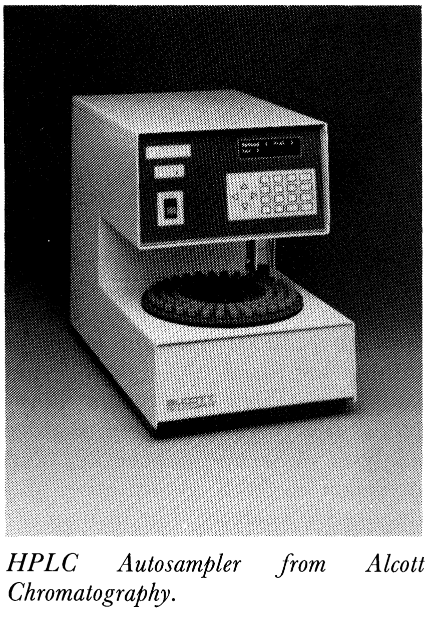

*HPLC Autosampler from Alcott Chromatography.*

Other features of the 738 include programmable, variable, injection volumes, pre-column derivatization and reagent dilution. Its vacuum fluorescent display provides instantaneous feedback of operating parameters in two-line dialogue. The 738 software allows on-the-fly programming, as well as priority-sample interrupt.

The Model 738 has both refrigeration and biocompatible options. Interfaces included on the unit are IEEE-488, RS-232-C and BCD.

*Alcott Chromatography, 5300 Oakbrook Parkway, #100 Nordoss, Georgia 30093, USA.*

### Refrigerated HPLC Autosampler
A refrigerated and heated HPLC Autosampler is available from Alcott Chromatography, Inc. Small and compact, the Model 728R can be temperature controlled from 4°C to 60°C and holds temperature to within ±1°C. Temperature control is maintained by a patented Peltier cooled/heated design, which eliminates completely the need for cumbersome water baths and coil cooling.

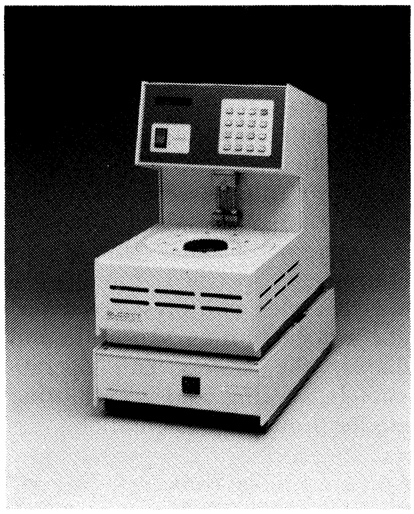

*Refrigerated HPLC autosampler from Alcott Chromatography.*

Designed for use with HPLC, ion chromatography or SFC systems, the 728R holds 64 samples and injects from 0·5 to 1000 μl of sample. The Model 728R uses no accessory gases and requires almost no maintenance. Its patented positive displacement design allows virtually no carry over and provides fail-safe interruption if sample injection does not occur due to blocked tubing. Each sample in the Model 728R can be individually programmed, and external control may be exercised through contact closures or through an RS-232-C portal. The model is also available in metal-free biocompatible versions.

*Alcott Chromatography (as above).*

### Three-detector HPLC system
Bacharach's HPLC system combines three of the most popular detection modes: UV, fluorescence, and conductivity. This single compact unit is designed for use in QC laboratories, screening, and teaching applications. The TriDet system features a trifunctional detector that allows one, two, or three of the detection modes to be monitored separately or simultaneously. The unit provides UV detection at 254 nm, senses fluorescence at 280 nm with excitation at 254 nm, and measures conductivity through the inlet and outlet disks of the cell.

*Bacharach, Inc., 625 Alpha Drive, Pittsburgh, Pennsylvania 15238, USA.*

### Data acquisition and control system
High pressure pump control for HPLC is provided with the ELAB PC-based data acquisition and control system. This system provides flexibility in control along with easy-to-use data acquisition, integration, automation, display, and calibration.

*OMS Tech, Inc., P.O. Box 430864, Miami, Florida 33243-0864, USA.*

### Chromatography data station
The DP900 fully automated chromatography data station was designed for GLP (Good Laboratory Practice). The instrument uses parameters of peak area, height, retention time, resolution, separation, capacity, and sensitivity to validate the performance of a single run. Groups of runs are checked by the RSD of these parameters as well as specific group statistics and correlation checks.

*ICI Instruments, 5 Lake Dr., Dingley, Victoria, Australia 3172.*

### HPLC system
A fully-automated, modular HPLC system, the Analyst, combines the ease of single keyboard control with the flexibility of multitasking

Windows-3 based software. The Analyst provides complete PC control and documentation of all system components. The system is available for either high or low pressure gradient applications with precise flow rate capability from 0·01 ml/min and UV detector sensitivity to 0·0005 aufs. The pumping options include the constaMetric 4100 quaternary gradient pump or the constaMetric 3500/3200 series isocratic pumps.

*LDC Analytical, 3661 Interstate Industrial Park Rd. N, P.O. Box 10235, Riviera Beach, Florida 33419, USA.*

### Preparative scale HPLC

The Versa Prep benchtop preparative scale HPLC offers solvent delivery capability of 20-260/40-390 ml/min at 4000 psig. The instrument has variable volume solvent mixing for up to four solvent gradients. Key features include sample recycle, fully automated operation via IBM PC, precolumn and multicolumn modules, 16 fraction collection ports, and packed column choices up to 3-in diameter. Adjustable volume high flow rate UV-VIS detector is standard. Other detectors can be used.

*Varex Corp., 4000 Blackburn Lane, Burtonsville, Maryland 20866, USA.*

### LABORATORY APPARATUS AND EQUIPMENT

### Pipette calibration system

The PCS pipette calibration system consists of an automated system that quickly and accurately verifies the actual volume delivered by mechanical action pipettes. The entire process takes less than 5 min per pipette, with no manual calculations required. The user pipettes ten individual reagent samplings into a single cuvette, and the device does the rest. The instrument automatically mixes the reagents in the cuvette, then measures, calculates, and prints a hard copy of test results for the laboratory's records.

*Artel, Inc., 12 Depot St., Windham, ME 04062, USA.*

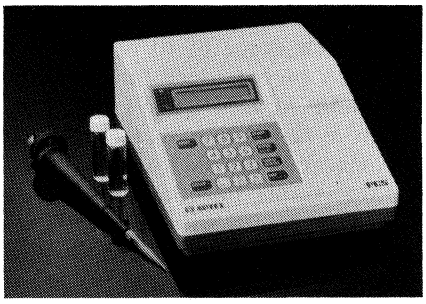

*The PCS pipette calibration system from Artel.*

### Test tube printer option

A DNF download file option for the TTP 20 test-tube printer offers simple and easy automation of a tedious manual procedure and virtually eliminates mislabelling. The option equips the unit to accept worklists of patient numbers, sample descriptions, accession numbers, and other data from a central database. Positive, detailed labelling of sample tubes is provided without the need to handle identification data a second time.

*BDC, 40 West Howard Unit 404, Pontiac, Michigan 48342, USA.*

### Tensiometer

The Lauda tensiometer automates time-consuming laboratory procedures such as CMC measurement. The instrument automatically measures and records liquid surface tension and interfacial tension at controlled and changing temperatures as programmed by the operator. De Nouy ring or Wilhelmy plate measuring devices can be used and continuous measurements taken without rupture of the lamella. Menu-driven software and automatic calibration make operation of the tensiometer simple.

*Brinkmann Instruments Inc., Cantiague Rd., P.O. Box 1019, Westbury, New York 11590-0207, USA.*

### Eight-channel viscosity system

The OctaVisc eight-channel viscosity measuring system, launched at the conference, automatically measures the viscosity of up to eight samples; and the companion Octa-Wash viscometer washing system automatically cleans and dries up to eight viscometers. Features include:

(1) Large dynamic range. Transparent or opaque samples ranging from 30 to 30 000 mm/s$^2$ at temperatures from 20 to 130°C, can be analysed.

(2) High sample throughput. Fifteen to 20 minutes are required to analyse eight samples. Eight viscometers can be cleaned and dried in 20 min.

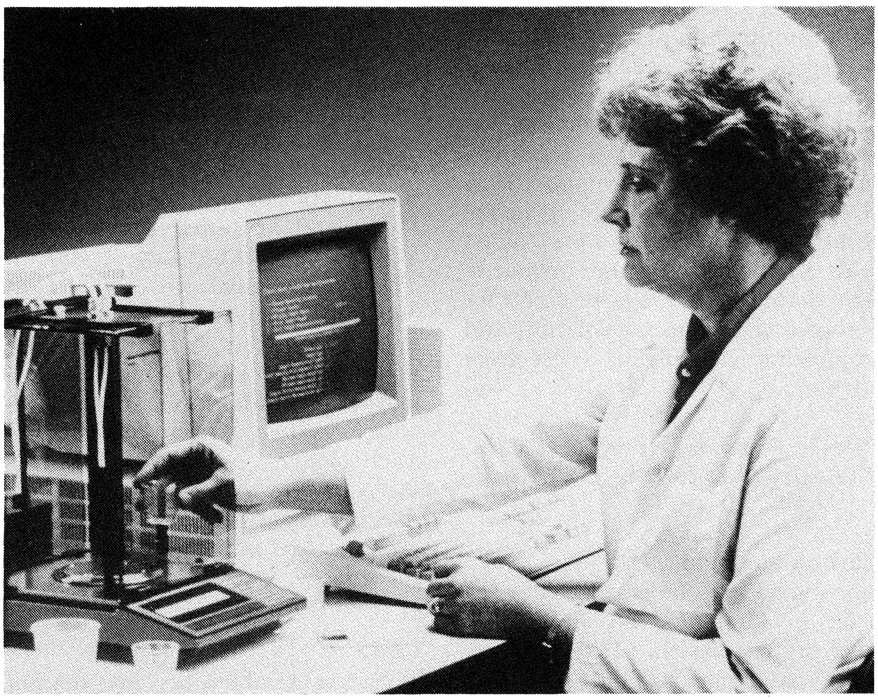

*The OstaVisc eight-channel viscosity measuring system.*

(3) High accuracy. Accuracy and precision is four to 10 times better than manual analysis.

(4) Viscometers are cleaned in a closed system, eliminating the dangers of solvent exposure and improving laboratory safety.

(5) Virtually any solvent or combination of solvents required for viscometry can be used in the system.

*Design Scientific, Inc., 4282 Pillsbury Road, Gainesville, Georgia 30507, USA.*

### SFA/FIA software

New windows software for the SFA/FIA analyser was released with auto pre- and post-dilutions, priority sample and sample addition during run, raw data storage, 16-channel simultaneous operation, statistics, and zoom. The modules feature automatic on-line digestion and distillation for phenols, cyanides, TOC, TP, and TKN. The SANplus automatic SFA/FIA system, OPA on-line process analyser, SP100 robotic BOD, COD, pH, conductivity and turbidity analyser, and block digester were also shown.

*Skalar USA, Inc., P.O. Box 579, Lilburn, Georgia 30226, USA.*

### Vapour pressure measurement

A portable Grabner CCA-VPS tester with multiple sampler automatically measures and reports the vapour pressure of up to six fuels, solvents, and similar volatile liquids at selected temperatures and pressures, using only a few ml of sample. ASTM RVP-Equivalent vapour pressure results are obtained in 5 min, along with total and absolute pressures. The device is particularly useful for compliance monitoring volatility and optimizing engine system performance.

*Petrolab Corp., 874 Albany Shaker Rd., Latham, New York 12110, USA.*

### Carbon analyser

The model 1505 laboratory analyser is for the automated measurement of total organic carbon (TOC), purgeable organic carbon (POC), and total carbon (TC) in water. The analyser operates accurately on a wide concentration range of 10 ppb to 2000 ppm by high temperature combustion and infrared detection of $CO_2$. With a 72-position integral sample sequencer (optional) and an automatic sample injection system, the user is provided with simplified unattended operation. The unit operates as a stand-alone analyser or can be interfaced for computer control.

*Ionics, Inc., Instrument Div., 65 Grove St., Watertown, Massachusetts 02172, USA.*

### Water analyser

The Aquatec system is an automated option for the routine testing of water. The unit simplifies high precision nitrogen, phosphorus, and chloride analysis in water. Based on interchangeable method cassettes, the device is designed for cost-effective analysis for ppb levels to ppm levels. The system can be operated either fully automated with an automatic sampler or semiautomatically. Preweighed chemical kits are available to guarantee correct dosage and to simplify reagent preparation.

*Tecator AB, c/o Perstorp Analytical Inc., 2875 C Towerview Rd., Herndon, Virginia 22071, USA.*

### LABORATORY COMPUTERS: SOFTWARE

### Relational Database System

The new family of laboratory information management systems is designed to use a relational database while running on a variety of hardware platforms (e.g. DEC, HP, and IBM) and operating systems (e.g. VAX/VMS, MPE-XL, and UNIX). The management systems, LIMS 3000, QC 3000, and Reference 3000, provide power and flexibility because they were developed using Ingres, a fourth generation, relational database system from Relational Technology.

*Advanced Systems Management, Inc., 1200 S. Acadian Thruway, Suite 110, Baton Rouge, Louisiana 70806, USA.*

### LIMS

Triton is a laboratory information management system (LIMS), available as a single-user system on an IBM 286 or compatible microcomputer, or as a multiuse system on an IBM 386 or compatible microcomputer or the IBM RISC System 6000. Features include: dictionary- or table-driven operation for application in any materials testing laboratory user-editable dictionaries/tables allowing for immediate additions of new test, methods, or instruments, direct instrument interface to the system, and remote printing.

*Caelus Systems Inc., 5 Spruce Park, Amherst, New Hampshire 03031, USA.*

### Batch Processor

PC LIMS is a laboratory information managment system for a wide variety of applications including pharmaceutical QC/R & D/stability studies, environmental control agencies, continuous/batch process industries, etc. Continuous development of the product has resulted in the introduction of the PC Lims Batch Processor for unattended automation of PC Lims tasks. Time-consuming jobs are automated, and instrument result files are automatically transferred to PC LIMS databases. Logging in of routine samples can be automated and samples can be logged in with information generated in other computers.

*L.I.M.S. Ltd., 419 S. Federal Hwy, Dania, Florida 33004, USA.*

### CHROMATOGRAPHY SOFTWARE

### Chromatography data management

The PYRAMID chromatography manager is a user-configurable data acquisition/analysis system. Running on Windows 3·0, the program offers user-friendly graphic programming/editing, high speed, and multitasking for other applications. The program's selectable GLP mode ensures total GLP-GMP compliance, with file audit trials and auto-naming. All functions can be configured for the exact components and applications in use, for simple operation. Optional interfaces control virtually all pumps, GCs, autosamplers, and detectors.

*Axxiom Chromatography, Inc., 11988 Challenger Ct., Moorpark, California 93021-7122, USA.*

### PC chromatography software

EZChrom software provides power and compatibility for IBM PCs while maintaining flexible, distributed data collection and processing for virtually all chromatography applications. Data may be collected from multiple Shimadzu Chromatopac chromatography processors in real time or from Chromatopac memory. Full parameter set up and data analysis can be accomplished independently at the computer or at the Chromatopac. The software operates under the Microsoft Windows 3·0 graphical environment, for ease of use.

*Shimadzu Scientific Instruments, Inc., 7102 Riverwood Dr., Columbia, Maryland 21046, USA.*

### Chromatography data system

Version 3·0 of the Chrom Perfect chromatography data system is PC-based software that can acquire data from Perkin Elmer-Nelson 700 and 900 series intelligent interfaces, in addition to Hewlett-Packard 3396 or 3393 integrators. Integrators and interfaces can be combined in the same system. The system Perfect offers extensive features for multi-tasking, integration, plotting, reporting, on-screen expansion and methods development.

*Justice Innovations, Inc., 465 El Capitan Pl., Palo Alto, California 94306, USA.*

### Chromatography software

CHROM/RTG is a comprehensive chromatography software package designed for GC, HPLC, or ion chromatography applications. The software combines real-time data collection, peak integration, report generation, and LIMS support into one easy-to-use package. A powerful, multi-instrument program, this program features a graphic, mouse-driven interface and real-time graphics (RTG) that run on all industry standard computer platforms, including UNIX, DOS/Windows 3, and OS/2.

*Labtech Corp., 400 Research Dr., Wilmington, Massachusetts 01887, USA.*

### Chromatography utilities

The HIPAC chromatography software package comprises a range of software tools to assist the development of HPLC separations. Designed to enable rapid and rational method development and optimization of isocratic reverse phase and normal phase binary mobile phases separations, HIPAC allows the user to incorporate computer assisted method development in a wide range of separation problems. Computer assisted optimization has advantages over the trial-and-error approach. HIPAC uses computer simulations, based on established chromatographic theory, which are reproducible and independent of the skills of the analyst.

*Phase Separations Inc., 140 Water St., Norwalk, Connecticutt 06854, USA.*

### Chromatographic data software

A powerful Macintosh tool for the acquisition, analysis, and display of chromatographic data, the PEAKS program, adheres to Apple's interface guidelines, and is easy to use. All acquisition parameters are automatic. The system hardware employs a 14-bit autoranging analogue to digital converter that provides 20 bits of effective resolution. All commonly used features such as baseline correction, injection times, and peak detection/integration are managed through the use of screen controls.

*Mac Science Solutions, 17 Water St., Milford, Massachusetts 01757, USA.*

### LIFE SCIENCES: INSTRUMENTATION

### Formalin recycling system

A formalin recycling system, the Pure-Form 2000, is designed for use in histology and pathology laboratories. The unit is fully automatic and reagent purity is ensured with the use of glass and PTFE design. Up to 5 gal of waste are loaded into the system, the user pushes a button, and the waste formalin is processed unattended in 12 h. All facets of the operation are microprocessor-monitored, including waste removal. The recovered formalin needs only to be buffered before it is reused.

*B/R Instrument Corp., P.O. Box 7, Pasadena, Maryland 21122, USA.*

### Multiple peptide synthesizer

The ACT model 350 robotic multiple peptide synthesizer enables rapid synthesis of up to 96 different peptides in one run. The instrument has an orbital shaker system to provide thorough resin agitation, and is controlled by interactive-window software that calculates solvent/reagent compositions and monitors synthesis progress. The Model 350 uses Fmoc chemistry and produces 5-75 mg of each peptide, and is suitable for screening methods and small-scale custom peptide production.

*Advanced ChemTech, P.O. Box 1403, Louisville, Kentucky 40201, USA.*

### Distillation system

The AutoMaxx 9000 automated distillation system can perform a broad range of distillations, from complete crude assays to fully automated analytical distillations. Maximum programming flexibility allows the operator to create a detailed program that can run a wide range of materials. A touch screen lets the operator view run parameters, a data table, or distillation curve. IBP or vapour pressure is diplayed in 5% increments when the test is underway.

*B/R International, Inc., P.O. Box 1026, Pasadena, Maryland 21122, USA.*

### LIQUID HANDLING APPARATUS

### Automated liquid dispenser

The MT200 sample distribution system uses liquid aspirating/dispensing modules to transfer samples and add reagents to the transferred samples. Available with one, two, or four independent probes, with independent liquid level detection, the MT200 can interface with up to eight external devices. The unit has sample barcode reading capability, and correlates information on barcodes, sample processing, and detector to provide reports. Multitasking allows the instrument to continuously communicate with mainframe computers for information transfer.

*Dynatech Laboratories, Inc., 1430 Sullyfield Circle, Chantilly, Virginia 22021, USA.*

### Electronic pipette

The METRON electronic pipette can perform the pipetting functions of many types of handheld pipettes. Microprocessor control together with state-of-the-art engineering provides for a highly precise liquid transfer instrument that is easy to use and ergonomically comfortable to handle. The pipette is also very adaptable to OEM applications where pipetting operations can be controlled by an alternate controller.

*Medical Laboratory Automation, Inc., 270 Marble Ave., Pleasantville, New York 10570-2982, USA.*

### MICROSCOPY, IMAGE ANALYSIS AND LABORATORY OPTICS

### Cell image analyser

The Samba 4000 cell image analyser for applications in the biological sciences features true colour analysis. Programs exist for a variety of research and clinical applications including ploidy, ER/PR, quantitative immunolabelling, morphometry, in situ hybridization, fluorescence, autoradiography, and others. A flexible open software environment is also available for the custom development of imaging programs.

*Dynatech Laboratories, Inc., 14340 Sullyfield Circle, Chantilly, Virginia 22021, USA.*

### Scanning electron microscope

The ABT-32X/II is a fully integrated digital scanning electron microscope and energy dispersive x-ray analysis system. The SEM can be operated from a simple push-button control panel or via a mouse and menu display on a SUN workstation. A computer-controlled stage is also available, which allows fully automated analysis sequences to be implemented.

*International Scientific Instruments, Inc., 6940 Koll Center Parkway, Pleasanton, California 94566, USA.*

### pH AND SPECIFIC ION ELECTRODES AND METERS

### Autochemistry system

The model 960 is a powerful, automated analytical instrument designed to enhance and expand measurements using ion-selective and pH electrodes. The instrument is intended for use in the QC laboratory where automation is needed to overcome problems of analytical complexity or sample throughput. Unlike other single parameter analysers or automatic titrators, this unit offers a wide range of techniques, options, and reagents for analysing species that can be measured by electrode, including species that cannot be titrated.

*Orion Research, Inc., The Schrafft Center, 529 Main St., Boston, Massachusetts 02129, USA.*

### SPECTROSCOPY AA

### Plasma source

A plasma source couples the high efficiencies of electrothermal atomization with plasma excitation resulting in the simultaneous detection of many elements at high sensitivity with few interferences. The source incorporates an integrated contact cuvette graphite furnace and an atmospheric pressure RF capacitively coupled plasma. The device can be adapted to function as an atomizer for atomic absorption and can be simply attached to most existing AA and AE instrumentation.

*Aurora Instruments, Ltd., 3031 Main St., Vancouver, British Columbia V5T 3G6, Canada.*

### Digestion system

The Microdigest M 301 modular digestion unit incorporates a focused microwave for rapid sample preparation before AAS and ICP analysis. The complete system consists of a control module that can control up to three units. Each unit can incorporate up to three reagent pumps. The digestion unit features the flexibility of a modular system, safety of automatic reagent addition, and reproducibility. The system eliminates acid excess by evaporating to dryness.

*Prolabo, 12, rue Pelee, BP 369, 75526 Paris Cedex 11, France.*

### FLUORESCENCE

### Multifrequency phase fluorometer

The K2 multifrequency phase fluorometer is designed for time-resolved fluorescence studies. It allows the resolution of multi-exponential decays, the anisotropy decay of fluorescence, time-resolved spectra, phase-resolved spectra, and phase-resolved kinetics. The device also works as a steady-state fluorometer for excitation and emission spectra, polarization spectra, synchronous luminescence spectra, and kinetics studies.

*ISS Inc., 309 Windsot Rd., Champaign, Illinois 61820, USA.*

### Luminescence spectrometer

The economical series 2 luminescence spectrometer offers both continuous and pulsed light sources for fluorescence and phosphorescence measurements. Capabilities include automated intracellular analyses using fluorescent probes for imaging or photometric measurements. The software package features a graphical user interface, multitasking, and automation.

*SLM-AMINCO, 810 W. Anthony Dr., Urbana, Illinois, USA.*

### FTIR

### Chemical process analyser

A full-scale chemical process analyser employs an infrared sensing probe immersed in a batch reaction vessel. The system, which typically collects and stores a complete mid-infrared spectrum once every 30 sec, measures the changes in individual chemical bonds as they take place, providing a window into the reaction. Multi-component analysis software is then used to provide time plots of the concentrations of each constituent in the reaction.

*Laser Precision Analytical/Analect, 17819 Gillette Ave., Irvine, California 92714, USA.*

### FTIR

The compact Analect Diamond-20 FTIR features one of the first applications of Microsoft Windows 3·0 to FTIR. The Windows-based software incorporates symbolic icons, interactive pop-up menus, and an on-line manual for help. The instrument's permanently aligned optics are hermetically sealed in a rugged cast body so that it can be operated in an uncontrolled environment without purging.

*Laser Precision Analytical/Analect (as above).*

### FTIR spectrometer

The Galaxy Series 7000 FTIR spectrometer provides high performance with 0·4 $cm^{-1}$ resolution capability and high sensitivity 18-bit ADC. With 17 user-selectable scan speeds, the instrument can be optimized for any analysis technique and detector combination. The Galaxy 7000 features fast detector change with computer-controlled selection of beam path and detector selection, and a sealed and desiccated interferometer to protect sensitive optical components.

*Mattson Instruments, 1001 Fourier Ct., Madison, Wisconsin 53717, USA.*

### Dedicated QC analysers

The 8200 Series QC analysers automatically analyse QC samples. One-button operation allows for high sample throughput with minimal sample preparation and operator training, and no operator variability. The 8210 liquids analyser system uses FTIR spectroscopy, accompanied by the ATR (attenuated total reflectance) technique. The 8210's optics provide significantly increased sensitivity over general-purpose instruments using standard sample compartments and standard sampling accessory designs. The 8210E FTIR analyser offers extended flexibility by allowing a variety of sampling accessories to be used while maintaining the same ease of operation.

*Nicolet Instrument Corp., Analytical Div., 5225 Verona Rd., P.O. Box 4451, Madison, Wisconsin 53711-0451, USA.*

### Research-grade IR microscopy system

The Nic-Plan combines advanced microscopic imaging with IR spectroscopy and chemical analysis when interfaced with a Nicolet FTIR spectrometer. The instrument features the View-Thru projected aperture masking system, which permits the entire sample image to be viewed while simultaneously positioning the apertures for IR sampling. The FTIR processor controls the IR beam path in the microscope. Computerised motor control is available for viewing/sampling, transmission/reflectance, and motorized stage positioning. The Nic-Plan has an on-axis optical design, Redundant Aperturing, and sample compensation with Cassegrainian Reflachromal optics for highly accurate sampling.

*Nicolet Instrument Corp. (as above).*

### Low-cost FTIR spectrometer

The Galaxy Series 3000 spectrometer is a low-cost, workhorse FTIR featuring 2 $cm^{-1}$ resolution with high stability optics. The instrument is compatible with any MS-DOS based IBM AT-compatible computer system. The Galaxy 3000 can be configured for dedicated analysis routines with FIRST macros, as a teaching system with U-FIRST, or as a full-featured analytical system with research level FIRST software.

*Mattson Instruments, 1001 Fourier Ct., Madison, Wisconsin 53717, USA.*

### FT-Raman spectroscopy system

A FT-Raman spectroscopy system is optimised to fully realise the intrinsic advantages of FT spectroscopy, making Raman spectroscopy applicable to a wider range of sample types. Two important advantages are the profound decreases in fluorescence and thermal decomposition effects. Additional advantages include the inherent throughput, multiplex, and wavenumber accuracy advantages, and the ability to collect high-resolution Raman spectra in a short time. The FT-Raman system provides capability for acquiring both FT-Raman and FTIR spectra on the same sample in only a few minutes. The NIC-Notch filter system performs measurements of Raman shifts very close to the Rayleigh line and simultaneous collection of Stokes and anti-Stokes data.

*Nicolet Instruments Corp. (as above).*

### ICP-AES

### ICP-AES spectrometer

The MAXIM series of high throughput, flexible, and high speed ICP-AES spectrometers increase laboratory productivity and analytical accuracy. The instrument offers simultaneous analysis with sequential flexibility, determining up to 60 elements for each analysis and access to a 200 wavelength array with full benefit of high resolution Echelle optics. The high-powered axial plasma source neutralizes interferences and other associated effects.

*Applied Research Laboratories/Fison Instruments, 24911 Avenue Stanford, Valencia, California 91355, USA.*

### Simultaneous plasma spectrometer

A new purged path optical design for the ICAP 61E simultaneous plasma spectrometer and PolyScan 61E simultaneous sequential plasma spectrometer has been introduced. The instruments can be used for elemental analysis of water, oils, and a wide variety of other materials. The purge path optical design can be used for the determination of elements in the vacuum ultraviolet wavelength region. The purge gas, normally argon or nitrogen, removes unwanted oxygen from the light path and environmentally isolates the optics from acid fumes in the laboratory.

*Thermol Jarrell Ash Corp., 8E Forge Pkwy., Franklin, Massachusetts 02038-9101, USA.*

### Sequential spectrometer

The ICP 2070 sequential spectrometer features complete integration of all components: FR generator, emission source, optics, electronics, cooling recirculator, vacuum pump, computer, monitor, printer, and optional sample changer. The system is a self-contained, ergonomically designed, sit-down work area. A solid state generator is microprocessor controlled to provide error-free plasma ignition, constant plume quality, and precise digital adjustment of output power.

*Baird Corp., 125 Middlesex Tpk., Bedford, Massachusetts 01730, USA.*

### Dual monochromator ICP system

The model D dual monochromator ICP system provides high quality analytical capabilities compared to other sequential ICP systems. As a result of a unique monochromator design, movement between lines is minimized with very accurate wavelength positioning. Direct drive to the desired wavelength is accomplished without time-consuming peak searches and associated positioning errors, particularly at analyte concentrations close to detection limit. The system is insensitive to temperature fluctuations.

*Spectro Analytical Instruments, Inc., 160 Authority Dr., Fitchburg, Massachusetts 01420, USA.*

### Sample introduction system for ICP

The Tandem dual multiplexed sample introduction system for ICP doubles the sample throughput of the PS series ICP/Echelle spectrometers. The system consists of two complete sample introduction systems (random access autosamplers, nebulizers, and spray chambers) and an inert shuttle valve, all operating under computer control. Available in glass and HF-resistant versions, the system can be used for any application requiring greater sample throughput such as environmental, geological, and lube oil wear metal analysis.

*Leeman Labs, Inc., 55 Technology Dr., Lowell, Massachusetts 01851, USA.*

### ICP/OES spectrometer

The Liberty 100/200 inductively coupled plasma optical emission sequential spectrometer offers very high resolution across the 160-900-nm spectrum with the use of a unique optical system. The Liberty 100 (air path) and 200 (vacuum path) models each have high-efficiency RF system for unequalled organics performance. Standard inert 'Sturman Masters' spray chamber runs all acids and organics. Central PC-controlled multitasking software allows complete operational flexibility and unattended operation. Accessories include a 500-position autosampler.

*Varian Associates, 200 Humboldt Court, Sunnyvale, California 94089, USA.*

### LC-MS

### Magnetic sector mass spectrometer

The LC/AX low-cost magnetic sector MS for LC-MS offers high sensitivity with better signal-to-noise and greater reproducibility than quadrupole mass spectrometers. Also on display will be the electrospray ionization source for use with the LC/AX. This instrument permits the analysis of high-molecular-weight proteins and polymers at high sensitivity: Existing JEOL spectrometers may be updated to use this technique.

*JEOL U.S.A., Inc., 11 Dearborn Rd., Peabody, Massachusetts 01960, USA.*

### MS

### Mass spectrometer

The Concept 32 is a high performance mass spectrometer designed for environmental, biotechnological, or general analytical applications. A basic two-sector instrument for use in research and general services work can be extended to a hybrid MS/MS or a four-sector MS/MS instrument with ultimate sensitivity and resolution. Resolutions ranging from 25 000 to 150 000 and mass ranges as high as 10 000 daltons at 8 Kv accelerating voltage are readily available.

*Dratos Analytical, 535 E. Crescent Ave., Ramsey, New Jersey 07446, USA.*

### Mass spectometer

The Automass mass spectometer, suited for high sensitivity analysis, features a quadrupole mass analyser with a rod diameter large than that found in other systems. Prefilters are used to eliminate quadrupole contamination, while Re-solver electronics give optimum peak shape. Ionization is achieved by a new electron impact source that provides ion transmission, sensitivity, and high resolution.

*Delsi Nermag, 98ter Bd. Helois, 95100 Argenteuil, France.*

### NMR

### FT-NMR spectrometer

The model R-1500 FT-NMR offers the following features: pulse Fourier transform data acquisition and processing for improved sensitivity, multitasking microcomputer control with post-run processing and disk storage to save time and increase throughput, 16k data points for maximum data acquisition and post-run processing resolution, deuterium lock for improved field stabililty, $T_1$ relaxation time via the inversion recovery method for complex spectral interpretation, and WEFT spectral processing to expand the dynamic range.

*Hitachi Instruments, Inc., 44 Old Ridgebury Rd., Danbury, Connecticut 06810, USA.*

### NMR spectrometer

Several data changes in the QE 300 NMR spectroscopy system provide faster and simpler operation, increase data handling capacity, and expand experimental capabilities. The enhanced system, QE Plus, includes: 3·5-in. floppy disk drive to increase data storage from 1 Mbyte to 1·44 Mbyte: 344 Mbyte hard disk drive as standard; 512K word memory as standard; addition of 256-step pulse programmer board; user ability to create individual H and C spectral libraries; and macro library doubled to 128. These macros contain such operations as calibration checks, routine H survey with integration, automated edited DEPT, 2D hetero correlation, and 2D phase-sensitive NOESY.

*GE NMR Instruments, P.O. Box 414, Milwaukee, Wisconsin 53201, USA.*

### Portable arc/spark emission spectrometer

The ARC-MET portable arc/spark emission spectrometer for alloy analysis and identification measures carbon in low alloy and tool steels down to 0·05%, as well as other elements commonly found in low alloy and stainless steels. The instrument can also perform identification via its 400 alloy library or internal grade tables. The 880 L Plus is an analytically advanced single/multi-stream on-line XRF analyser that is capable of measuring up to six elements in each of six streams.

*Outokumpu Electronics Inc., 860 Town Center Drive, Langhorne, Pennsylvania 19047, USA.*

### TITRATION APPARATUS

### Equilibrium Titrator

The Mitsubishi model GT-06 microprocessor-controlled equilibrium titrator has a built-in printer that displays both the classical titration curve as well as the first derivative, data, results, and calculations. All parameters, calculations, and sample information can be easily programmed by the user. Demonstration files stored in permanent memory contain parameters for common titration methods. The demonstration files can be loaded into active memory or used directly, or can be simply amended by the operator for customised titration methods. The model GT-06 can automatically operate up to four burettes, and connect to a balance, a sample changer, a full-size graphics printer, or a solvent dispenser.

*Cosa Instruments Corp., 55 Oak St., Norwood, New Jersey 07648, USA.*

### Automatic sample changer

The model CHG-310 automatic sample changer for automatic titration analysis can accommodate either 12 or 18 samples using standard beaker sizes. The unit has predefined sampling sequences and can also be user-programmed for difficult non-standard titration sequences. A unique electrode washing system allows for aqueous and solvent rinsing control. Optional accessories include a controlled water bath, fumehood, multiple stirring, and a conical flask turntable.

*Kyoto Electronics Manufacturing USA, Inc., 2 Edison Place, Springfield, New Jersey 07081, USA.*

### PC-based titration system

The PC-Tritrate IBM PC-based system for acid/base titrations allows control over titrations and provides quality data analysis. Features include; post-run analysis, specialised database, a scheduler that allows user to define complex sequences before, during, and after titrations, parser that permits the entry of up to 18 equations, conventional data, and Grans's analysis.

*Man-Tech Associates, Inc., 900 Hertel Ave., Buffalo, New York 14216-0209, USA.*

### Karl Fischer titrator

The DL35 volumetric Karl Fischer titrator features methods storage, statistics calculations, selectable result units, and on-line background rift monitoring and compensation. Ten routine procedures are supplied, which also lets the user store up to 50 methods for moisture measurements. The titrator's design assures minimal background moisture intrusion, thereby allowing low level analyses of water in analytical mixtures or products. For applications where the sample cannot be introduced directly in the Karl Fischer reagent, two optional evaporation ovens are available.

*Mettler Instrument Corp., Analytical Instrument Corp., Analytical Instruments Div., Box 71, Princeton-Hightstown Rd., Hightstown, New Jersey 08520, USA.*

### Karl Fischer coulometer

The DL37 KF Coulometer precisely determines very low levels of moisture in solid, liquid, and gaseous samples. The DL37 KF, with matching DO337 drying oven, complements the company's Karl Fischer titrators when water content is to be determined in the microgram range. Compact and highly automated, the DL37 allows five user-defined methods to be stored and recalled via its method key.

*Mettler Instrument Corp. (as above).*

### Karl Fischer titrators

A complete line of Karl Fischer titrators set new standards for accuracy, performance, and operating convenience in moisture detection. Using the latest in electronic technology, advanced chemistry, and precision engineering, these titrators offer many advantages in the detection of moisture in a wide variety of solid, liquid, and gaseous materials.

*Orion Research, Inc., 529 Main St., The Schrafft Bldg., Boston, Massachusetts 02129, USA.*

### UV-VIS

### Diode array spectrophotometer

The programmable DU 7500 diode array spectrophotometer is designed for life science laboratories, including research laboratories, where scientists work with microvolume and ultramicrovolume samples as small as 5 μl. Typical applications include the identification and characterization of enzymes, proteins, and nucleic acids. The DU series 7000 instruments feature FSQ full spectrum quantitation, which allows researchers to determine in concentration units the individual components in complex mixtures.

*Beckman Instruments, Inc., 2500 Harbor Blvd., Fullerton, California 93634-3100, USA.*

### UV-VIS spectrophotometer

The model GBC 916 double-beam UV-VIS spectrophotometer features a complete range of automated accessories. The instrument is controlled by an external computer that offers full colour graphics and complete data storage. The operating software provides a fully integrated system for instrument control, data analysis, and report generation. An extensive range of graphics manipulation functions, including 3-D display, is provided.

*GBC Scientific Equipment Pty. Ltd., 22 Brooklyn Ave., Dandenong, Victoria 3175, Australia.*

### Near UV-VIS spectrophotometer

The model S 500 near UV-VIS spectrophotometer measures wavelengths between 330 and 900 nm. The unit has a microprocessor and features: automatic adjustment of monchromator; software for reading transmittance, absorbance, multistandard concentration, kinetics, and spectrum; and storage of analytical methods. The instrument has a cell compartment for cells 10 to 40 mm long and can be equipped with a variety of accessories including a funnel flow-through cell, a 30 μl flow-through cell, and a graphic printer.

*Secomam, 9, rue de l'Escouvrier-Z.I., F-95200, Sarcelles, France.*

### PC-controlled spectrophotometer

The model S.1000PC UV-VIS spectrophotometer features a monochromator that is directly controlled by a PC. The instrument has a language that combines spectrophotometer controlling commands with general microcomputer language instruction. The basic software incorporates a comprehensive set of instructions. The instrument can be used as an online monitor and automated for drug dissolution testing, or an integrator detector for chromatography.

*Secomam (as above).*

### Spectrophotometer

The PRIM.C features original grating optics, wide wavelength range, and chopped optics to prevent drifting of zero of T. Advanced electronics feature automatic zero and extended software programs. Switching the power supply reduces overheating and allows independent battery operation. The keyboard and menus are shown on LED display for easy selection of absorbance, transmittance, concentration, and kinetics modes. The unit also features a wavelength range of 330-990 nm.

*Secomam (as above).*

### X-RAY SPECTROSCOPY

### WDXRF sequential spectrometer

The model 8410 WDXRF sequential spectrometer is a small, versatile unit that meets the needs of any laboratory, including petrochemical, plastics, manufacturing, academic, or corporate research. Standard features include the moire fringe (gearless) goniometer, three collimators, nine crystal positions, encoded cassettes for automated sample handling, end window RH x-ray tube for maximum excitation, and Microsoft Windows driven software for maximum flexibility.

*Applied Research Laboratories/Fisons Instruments, 24911 Avenue Stanford, Valencia, California 91355, USA.*

